# Expression of Codon-Optimized Plant Glycosyltransferase *UGT72B14* in *Escherichia coli* Enhances Salidroside Production

**DOI:** 10.1155/2016/9845927

**Published:** 2016-08-15

**Authors:** Feiyan Xue, Huili Guo, Yingying Hu, Ran Liu, Lina Huang, Heshu Lv, Chunmei Liu, Mingfeng Yang, Lanqing Ma

**Affiliations:** ^1^Key Laboratory of Urban Agriculture (North), Ministry of Agriculture, Beijing 102206, China; ^2^College of Biological Science and Engineering, Beijing University of Agriculture, Beijing 102206, China; ^3^Beijing Collaborative Innovation Center for Eco-Environmental Improvement with Forestry and Fruit Trees, Beijing 102206, China

## Abstract

Salidroside, a plant secondary metabolite in* Rhodiola*, has been demonstrated to have several adaptogenic properties as a medicinal herb. Due to the limitation of plant source, microbial production of salidroside by expression of plant uridine diphosphate glycosyltransferase (UGT) is promising. However, glycoside production usually remains hampered by poor expression of plant UGTs in microorganisms. Herein, we achieved salidroside production by expression of* Rhodiola* UGT72B14 in* Escherichia coli* (*E. coli*) and codon optimization was accordingly applied.* UGT72B14* expression was optimized by changing 278 nucleotides and decreasing the G+C content to 51.05% without altering the amino acid sequence. The effect of codon optimization on UGT72B14 catalysis for salidroside production was assessed both* in vitro* and* in vivo*.* In vitro*, salidroside production by codon-optimized UGT72B14 is enhanced because of a significantly improved protein yield (increased by 4.8-fold) and an equivalently high activity as demonstrated by similar kinetic parameters (*K*_M_ and *V*_max_), compared to that by wild-type protein.* In vivo*, both batch and fed-batch cultivation using the codon-optimized gene resulted in a significant increase in salidroside production, which was up to 6.7 mg/L increasing 3.2-fold over the wild-type* UGT72B14*.

## 1. Introduction

Glycosylation of small molecules has a profound impact on their biological properties including bioactivity, solubility, and stability. The majority of glycosylation reactions in nature are mediated by glycosyltransferases, which comprise a large and divergent polyphyletic multigene superfamily [1, 2]. In plants, glycosylation catalysed by uridine diphosphate (UDP) glycosyltransferases (UGTs) [2–4] is involved in the synthesis of many secondary metabolites. Studies on the recombinant expression of UGTs have resulted in the production of various plant secondary metabolites as glycosides [2, 5, 6]. However, many valuable plant glycosides remain difficult to produce in high yield and in a timely manner.

Microbes, especially* Escherichia coli *and* Saccharomyces cerevisiae*, can be engineered to produce plant natural products by introducing key enzymes [7, 8].* E. coli *is the most widely used, well-characterized heterologous host and proved to result in more products yield than* S. cerevisiae *[4, 9, 10]. Notably, poor expression of wild-type plant genes in* E. coli* has restricted the yield of products [11, 12]. One strategy for overcoming this limitation is codon optimization [12–14].

**Figure 1:**
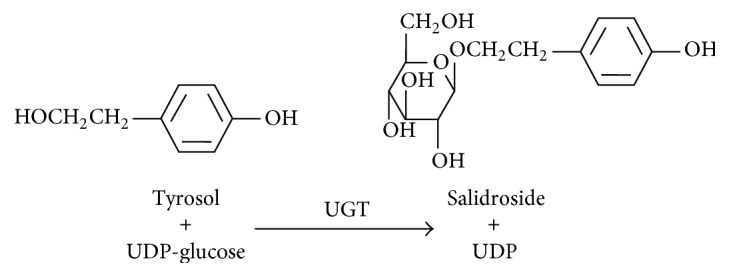
Reaction catalysed by UGT during salidroside production.

Salidroside (8-*O*-*β*-d-glucoside of tyrosol) is one of the main plant secondary metabolites in* Rhodiola* and has been demonstrated to have adaptogenic and ergogenic capacity [15]. Consequently, demand for salidroside compounds has grown, but* Rhodiola* has a long growth period and low salidroside content, and an alternative method of salidroside production is thus needed. Glycosylation typically occurs as one of the last steps during natural product synthesis [5], and this is the case for salidroside [16, 17]; the last step involves glycosylation of tyrosol, catalysed by UGT (Figure 1). In our previous work, three different UGT genes,* UGT72B14*,* UGT73B6*, and* UGT74R1*, were isolated from* Rhodiola sachalinensis* and characterized [16, 17]. Among the three UGTs, UGT72B14 demonstrated the highest activity for salidroside production, with a catalytic efficiency (*V*_max_/*K*_M_) of 12.3, which was 620% and 180% higher than UGT74R1 and UGT73B6, respectively [17]. Accordingly, we investigated the effect of codon optimization on the heterologous expression of* UGT72B14* in* E. coli* and assessed the impact on salidroside production in the present study.

## 2. Methods

### 2.1. Strains and Plasmids

The host* E. coli* (DE3) strain was from a frozen stock stored at the Key Laboratory of Urban Agriculture (North) of the Ministry of Agriculture of China, Beijing, China. This strain was used for salidroside production following transformation with the appropriate plasmid. The pET-28a (+) plasmid was purchased from Novagen (Darmstadt, Germany) and used for cloning and expression of* UGT72B14* in the* E. coli* host strain.

### 2.2. Codon Optimization

The wild-type* UGT72B14* gene was isolated from* Rhodiola sachalinensis *(GenBank Accession Number EU567325) and optimized by replacing codons predicted to be less frequently used in* E. coli* with more favored codons, according to the Codon Usage Database and graphical codon usage analyzer. The optimized gene with introduction of restriction sites (*Nco* I and* Xho* I) was synthesized by Inovogen Tech. Co. (Beijing, China).

### 2.3. Protein Expression

The synthetic gene was digested and ligated into the prokaryotic expression vector pET-28a (+) at the same restriction sites (*Nco* I and* Xho* I), under the control of T7 promoter. The resulting plasmid was transformed into the* E. coli* host. Following identification of a positive transformant, cells were grown at 37°C with shaking at 180 rpm in LB (Luria-Bertani) medium containing 50 *μ*g/mL kanamycin. When the desired OD_600_ was reached, the temperature was reduced to 20°C, shaking was slowed to 120 rpm, and expression was induced with isopropyl *β*-d-1-thiogalactopyranoside (IPTG) at a final concentration of 0.8 mM. Cultures were harvested at various time points to measure OD_600_ and do sodium dodecyl sulfate-polyacrylamide gel electrophoresis (SDS-PAGE) analysis. Cell pellets, obtained by centrifugation at 12,857 ×g for 5 min, were resuspended in sterile H_2_O and subjected to SDS-PAGE analysis.

### 2.4. Protein Purification and Measurement of Enzyme Activity

Recombinant* E. coli* cultures were incubated at 37°C with shaking at 180 rpm in LB medium containing 50 *μ*g/mL kanamycin. When the OD_600_ reached 0.3 ± 0.1,* E. coli* harbouring the optimized* UGT72B14* plasmid was induced at 20°C with shaking at 120 rpm as described above for a further 6–8 h. Cell pellets were harvested by centrifugation (8,000 ×g; 10 min; 4°C) and resuspended in lysis buffer (20 mM Tris, pH7.5, 300 mM NaCl, 20 mM imidazole, 1 mg/mL leupeptin, 2 mg/mL aprotinin, and 100 mg/mL lysozyme). Cells were disrupted by sonication (30 × 3 s) with ice bath. Supernatant was separated from the sonication homogenate by centrifugation (10,000 ×g; 10 min; 4°C) and passed through Ni-NTA His-Bind*™* Resin (Novagen, Madison, WI, USA) column containing Ni^2+^ as an affinity ligand. After washing with elution buffer (20 mM Tris, pH7.5, 300 mM NaCl, and 20 mM imidazole), unbound contaminant proteins were removed. His-tagged proteins were eluted with washing buffer (20 mM Tris, pH7.5, 300 mM NaCl, and 300 mM imidazole) and purified using a PD-10 column (Amersham Pharmacia Biotech, Uppsala, Sweden) and then concentrated by a centrifugal filter unit (YM-50 kD, Millipore). The efficiency of purification was monitored by SDS-PAGE analysis. The concentration of purified UGT72B14 was determined using BCA protein assay kit (Sigma-Aldrich, USA), and then the purified protein yield was assessed by calculating the purified protein content per litre of culture broth. For recombinant* E. coli* harbouring the wild-type* UGT72B14* plasmid, induction was initiated at OD_600_ = 0.7 ± 0.1 and growth continued for a further 9 h. Cells were then harvested and protein was purified as described above. To measure the kinetic parameters, tyrosol (Sigma-Aldrich, Saint Louis, Missouri, USA) and UDP-glucose substrates were incubated with purified protein at 30°C for 30 min, and the salidroside product was identified using High Performance Liquid Chromatography (HPLC). Enzyme assays were carried out as previously described [17].

### 2.5. Measuring Salidroside Production in the Recombinant* E. coli* Strains

Cultures were grown and induced as described above, and tyrosol, glucose, and sodium citrate (all analytical grades) were added in equimolar concentrations and culturing continued at 30°C with shaking at 150 rpm. Samples were harvested at various time-points during fermentation by centrifugation at 12,857 ×g for 10 min. Supernatants were analyzed directly by HPLC or freeze-dried and extracted with an equal volume of ethanol, evaporated, and dissolved in methanol to prepare Liquid Chromatography and Mass Spectrometry (LC-MS) samples as described previously [17].

## 3. Results 

### 3.1. Codon Optimization and Its Effect on Protein Expression

UGT72B14 was found to have the highest activity for salidroside production and was thus considered the ideal candidate for testing the effect of codon optimization. The full-length gene sequence (1671 bp) has a G+C content of 56.75% and includes an open reading frame (ORF) of 1422 bp that encodes a polypeptide of 473 amino acids [17]. Codons such as AGA (Arg), AGG (Arg), CGA (Arg), CGG (Arg), AUA (Ile), CUA (Leu), CUC (Leu), and CUU (Leu), present in the wild-type plant sequence, are used at less than 10% in* E. coli*. Bioinformatics analysis was performed to identify codons that could be modified to better match the host codon usage preferences without altering the resulting amino acid sequence. We replaced most of them with those that are more frequently used in* E. coli* BL21 (DE3). Codons encoding Arg, Gly, and Leu were altered in 93%, 67%, and 81% of cases, respectively. The termination codon was also modified as UAA. It is well documented that ORFs containing high G+C content are often poorly expressed in A+T rich hosts [14, 18]. After altering 278 nucleotides, the G+C content was decreased to 51.05% in the optimized sequence. The codon-optimized UGT72B14 was submitted to NCBI (GenBank Accession Number KU523897).

**Figure 2:**
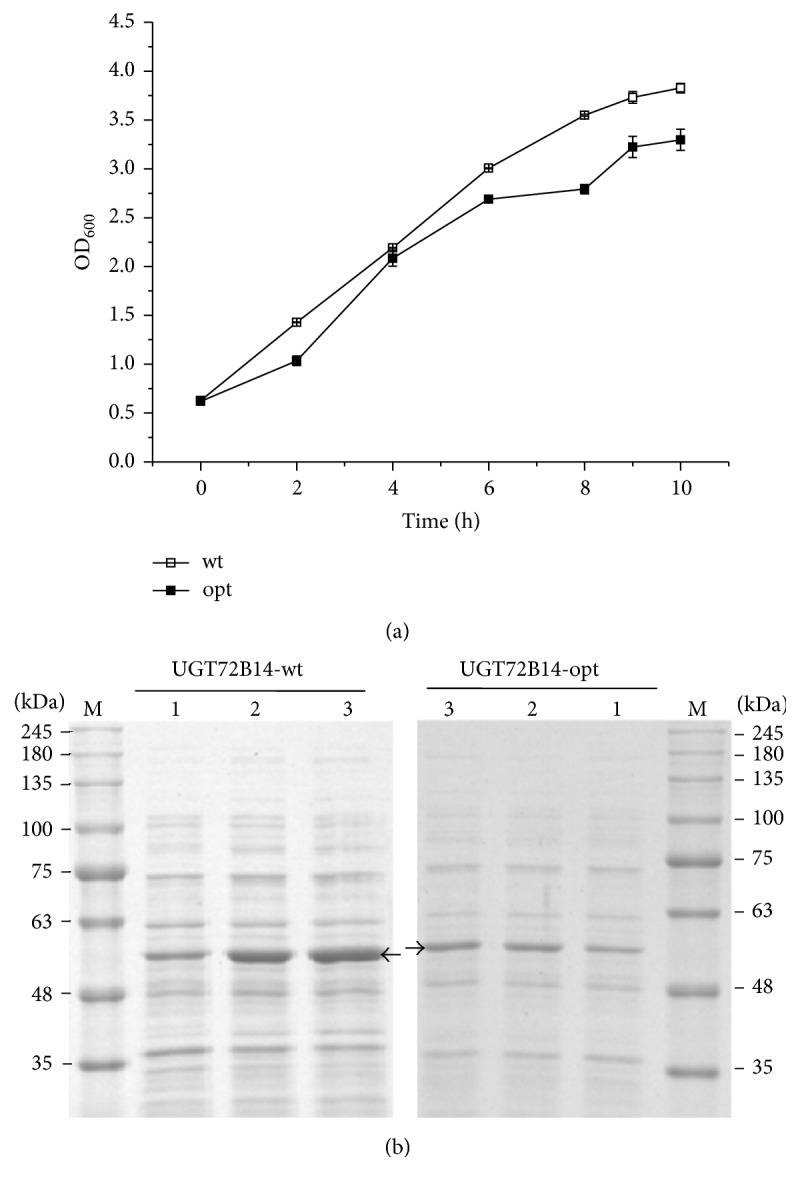
Growth profile and protein expression of the recombinant strains harbouring codon-optimized and wild-type* UGT72B14* gene. (a) Cell growth of recombinant strains. Data are presented as mean of three biological replicates, and error bar represents the standard deviation (SD). (b) SDS-PAGE analysis of protein expression. Sample of 20 *μ*L was used for SDS-PAGE analysis. UGT72B14-opt = codon-optimized UGT72B14; UGT72B14-wt = wild-type UGT72B14. Lane M: protein molecular weight markers. Lanes 1–3: protein expression at 4, 6, and 9 h after induction. Target protein is indicated by arrows.

**Figure 3:**
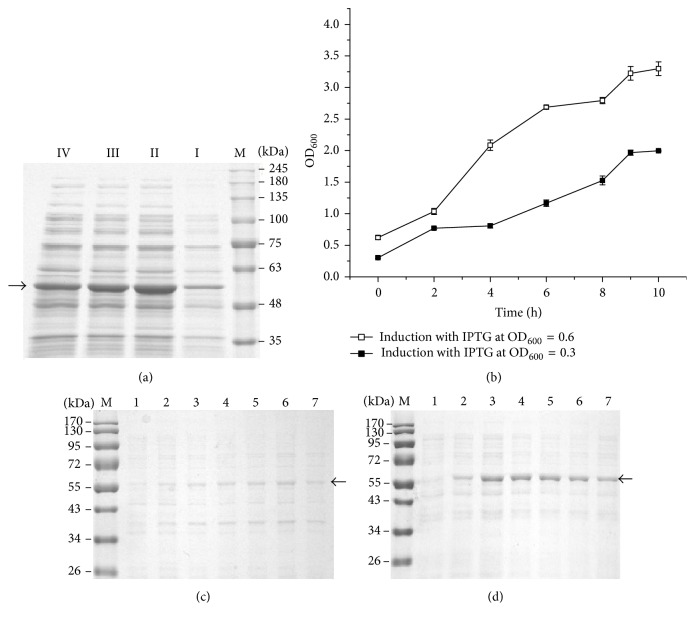
Optimization of UGT72B14-opt expression. (a) Protein expression at 10 h after induction with IPTG at varying OD_600_. (b) Growth profile of recombinant strain during induction. (c) Time courses of protein expression with IPTG induction at OD_600_ = 0.621 ± 0.032. (d) Time courses of protein expression with IPTG induction at OD_600_ = 0.303 ± 0.011. Lane M: protein molecular weight markers. Lanes I*–*IV: UGT72B14-opt expression at OD_600_ ~ 0.1, 0.3, 0.6, and 0.9 with 10 h after induction. Lanes 1–7: UGT72B14-opt expression at 0, 2, 4, 6, 8, 9, and 10 h after induction. Cell pellets were resuspended, the OD_600_ was adjusted to 1.00 ± 0.02, and 15 *μ*L was used for SDS-PAGE analysis. Target protein is indicated by arrows. Data are presented as mean of three biological replicates, and error bar represents the SD.

To study the effect of codon optimization on UGT72B14 expression, the optimized gene was synthesized and used to construct the recombinant plasmid that was subsequently transformed into* E. coli*, and protein expression was assessed by SDS-PAGE. The cell density at the time of IPTG induction is believed to be important for protein expression, and an optical density of 600 nm (OD_600_) of 0.6–0.8 was previously demonstrated to be optimum for expression of both UGT73B6 and UGT72B14 [16, 17, 19]. This cell density was thus used for induction of expression of the optimized* UGT72B14* gene in the present study. Considerable cell density (Figure 2(a)) and maximum accumulation of the target protein 9 h after induction (Figure 2(b)) were observed, and the variation of cell growth and protein expression was consistent with our previous work [17]. However, expression levels of the codon-optimized* UGT72B14* were even lower than the wild-type gene when induction was performed at OD_600_ ~ 0.6 (Figure 2(b)). Induction was thus tested at OD_600_ ~ 0.1, 0.3, 0.6, and 0.9, and OD_600_ ~ 0.3 was found to result in the highest expression of the codon-optimized* UGT72B14* gene (Figure 3(a)). SDS-PAGE analysis showed that induction at OD_600_ ~ 0.3 and growth for a further 6–8 h resulted in peak target protein levels (Figure 3(d)), which was overall significantly superior to that induction at OD_600_ ~ 0.6 (Figure 3(c)), though lower in cell growth (Figure 3(b)). To our best knowledge, this interesting result is firstly observed in* Rhodiola *UGT expression. However, it is consistent with a previous report of other proteins [20] that IPTG addition decreased cell growth but enhanced expression of the target gene when added at a relatively low cell density (OD_600_ ~ 0.3).

### 3.2. Effect of Codon Optimization on Salidroside Production* In Vitro*

**Figure 4:**
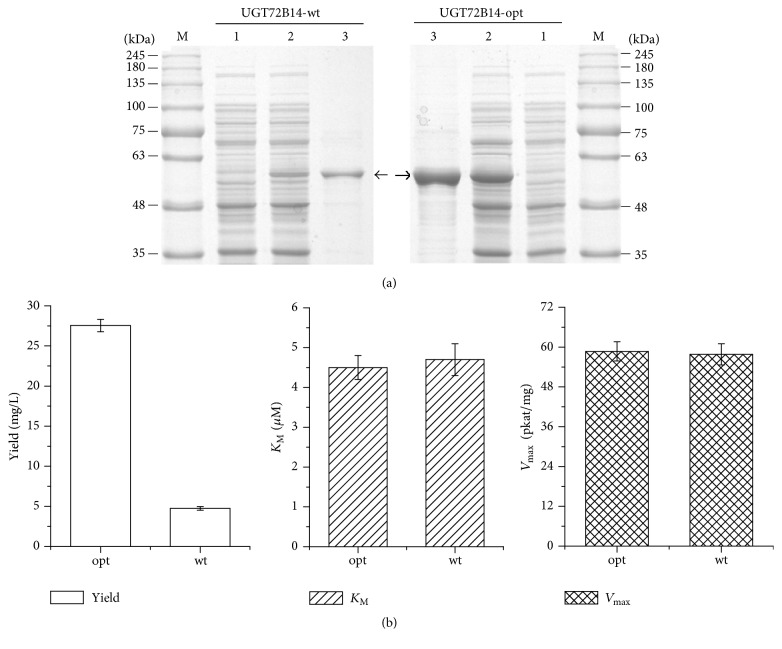
Result of the recombinant protein yield and salidroside production* in vitro*. (a) SDS-PAGE analysis of the recombinant protein expression and purification. Lane M: protein molecular weight markers. Lane 1: total protein in uninduced strain. Lane 2: total protein in induced strain. Lane 3: concentrated protein after being purified by Ni affinity chromatography and PD-10 column. Sample of 15 *μ*L was used for SDS-PAGE analysis. Target protein is indicated by arrows. (b) Result of salidroside production catalysed by UGT72B14* in vitro*. Protein yield data of the optimized UGT72B14 was obtained by purified protein after induction of 7 h initiated with OD_600_ = 0.325 ± 0.021 while that of the wild-type UGT72B14 was obtained by purified protein after induction of 9 h initiated with OD_600_ = 0.632 ± 0.031. The recombinant enzyme catalysis reaction system (100 *μ*L) contained 50 mM Tris-HCl (pH 7.5), 2 mM UDP-glucose, 250 *μ*M tyrosol, and the enzyme protein of 0.2 mg, proceeded for 30 min at 30°C, and terminated with 200 *μ*L MeOH. Data were presented as mean ± SD.

To examine whether codon-optimized UGT72B14 are capable of converting tyrosol to salidroside, the His-tagged UGT72B14 recombinant proteins were purified using Ni affinity chromatography. Result of SDS-PAGE analysis indicated that the codon-optimized gene resulted in not only more target protein expression but also more efficient purification (Figure 4(a)). It was 27.55 mg/L of purified protein obtained from the culture of strain harbouring the codon-optimized* UGT72B14*, which was an increase of 4.8-fold over that from the wild-type gene (Figure 4(b)). To investigate the effect of codon optimization on salidroside production* in vitro*, the activity of codon-optimized and wild-type UGT72B14 protein was tested using tyrosol and UDP-glucose as substrates, and production of salidroside was monitored by HPLC and LC-MS. There was no significant difference in specific activity between codon-optimized and wild-type enzymes, as demonstrated by comparable kinetic parameters (*K*_M_ and *V*_max_; Figure 4(b)). Wild-type UGT72B14 has been already proved to exhibit highest level of activity for salidroside production, compared to the other two UGTs we have cloned [17]. Here, an equivalently high enzyme activity and a significant improvement in protein yield of codon-optimized UGT72B14 will offer great promise for salidroside production* in vitro*.

### 3.3. Effect of Codon Optimization on Salidroside Production* In Vivo*

**Figure 5:**
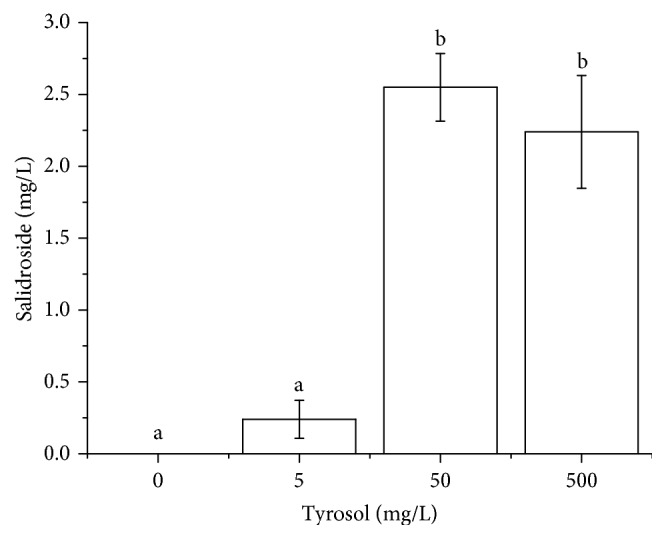
Effect of tyrosol concentration on salidroside production in* E. coli* expressing codon-optimized UGT72B14. Data are presented as mean of three biological replicates, and error bar represents the SD. Different letters above bars indicate a significant difference at *p* < 0.05 according to Duncan's test.

**Figure 6:**
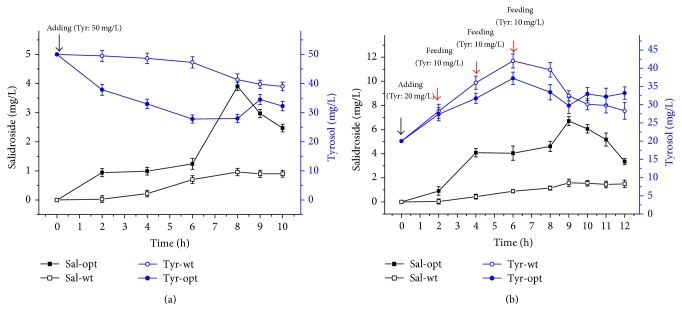
Result of salidroside production* in vivo*. (a) Batch cultivation. (b) Fed-batch cultivation. Sal-opt and Sal-wt refer to salidroside synthesized by strains harbouring codon-optimized and wild-type* UGT72B14* plasmids, respectively. Tyr-opt and Tyr-wt refer to tyrosol concentration in the culturing broth of strains harbouring codon-optimized and wild-type* UGT72B14* plasmids, respectively. Black arrows indicate substrate additions, and red arrows indicate substrate feeding during cultivation. Samples were tested three times. Data are presented as mean, and error bar represents the SD.

Utilizing recombinant enzymes to synthesize plant secondary metabolites in* E. coli* has many advantages, including simple nutritional requirements, easy cultivation, and the ability to prevent enzyme inactivation [6, 7, 21, 22]. The plant secondary metabolite salidroside is synthesized via a one-step glycosylation reaction from tyrosol in* Rhodiola *[16, 17, 23]. Following confirmation of codon-optimized UGT72B14 expression and catalytic activity* in vitro*, substrates were added to growing cultures at 6 h after induction with IPTG and conversion of tyrosol to salidroside was investigated. Tyrosol was tested at 0, 5, 50, and 500 mg/L, and most salidroside was observed at 50 mg/L tyrosol (Figure 5). This tyrosol concentration was subsequently used for a time-course experiment on cultures harbouring codon-optimized or wild-type* UGT72B14* plasmids. As shown in Figure 6(a), salidroside was first detected at 2 h after addition of tyrosol, and the concentration gradually increased, peaked at 8 h, and then declined over the following 2 h. A similar pattern was observed for both recombinant strains, but the codon-optimized culture exhibited a much wider range. Codon optimization increased the maximum salidroside production by 3-fold. Time-course experiments revealed a tendency for the target protein yield to decline at the end of batch fermentation, which was consistent with previous reports [18, 24]. We tentatively conclude that batch cultivation resulted in substrate inadequate utilization at the early stages of fermentation, and the low product yield encouraged us to investigate other ways of improving salidroside production.

Fed-batch cultivation has been shown to be an effective way to enhance the expression of target proteins and the yield of target products in* E. coli* [25–28]. In the present study, we performed fed-batch experiments by feeding cultures with four additions of 50 mg/L tyrosol (an initial addition of 20 mg/L, and three more additions of 10 mg/L at 2 h intervals). In these experiments, salidroside was first detected in the culture broth at 2 h after the first tyrosol addition and was then maintained at a high level (with slight fluctuations) between 4 and 11 h after the first addition (Figure 6(b)). Up to 6.7 mg/L of salidroside was accumulated at 9 h in the codon-optimized culture, which was an increase of 3.2-fold over the culture harbouring the wild-type* UGT72B14* plasmid. Compared with salidroside production in the batch cultivation method, fed-batch fermentation achieved a higher titre, and salidroside production was stable over a longer period. A gradual supplement of tyrosol is thus beneficial for salidroside production, and optimizing the amount of tyrosol added could further increase salidroside yield. This may explain the higher yield of salidroside that was achieved following reconstitution of the tyrosol biosynthetic pathway in* E. coli* [19].

## 4. Discussion

Glycosyltransferases have proved to be very important for plant secondary metabolism synthesis and have become an important research field in improving catalytic efficiency [3, 29]. In terms of UGTs, they have emerged as promising catalyst for UDP-sugar based glycosylation focusing on small molecules like salidroside [4]. Although many UGTs have been cloned and expressed in* E. coli* glycoside production remains hampered by poor expression of plant genes in this prokaryote host [11]. Codon optimization, as an effective approach to improving expression of heterologous protein, has attracted considerable attention and was applied successfully in some cases [12–14, 17]. However, no systematic studies of codon optimization have been reported for plant UGTs.

The UGTs of* Rhodiola sachalinensis* as one of the most important plants for salidroside supply [30, 31] thus aroused our great interest. Three* Rhodiola* UGTs were firstly cloned and characterized in our previous work [17]. With the sequence of* UGT73B6*, one of the three UGTs we published, salidroside has been synthesized* in vivo* by Bai et al. [19]. Here with the sequence of* UGT72B14*, codon optimization was systematically studied and proved favorable for enhancing salidroside production both* in vivo* and* in vitro*.

## 5. Conclusions

The optimized* UGT72B14* expression at a high level was benefited from induction with IPTG at a reasonably low cell density (OD_600_ ~ 0.3). A fed-batch cultivation method proved optimal for salidroside production in terms of overall yield and the duration over which maximum production occurred. As demonstrated favorable for enhancing* UGT72B14* expression and salidroside production in a timely manner in* E. coli*, codon optimization therefore deserved to be used to improve plant* UGT*s expression in heterologous hosts to enhance their corresponding glycoside production.
